# Epidemiology, clinical characteristics and risk factors of COVID-19 among children in Saudi Arabia: a multicenter chart review study

**DOI:** 10.1186/s12887-021-02959-8

**Published:** 2022-02-12

**Authors:** Ahmad AlGhamdi, Yousef Al Talhi, Abeer Al Najjar, Ahmed Sobhi, Alaa Al Juaid, Amany Ibrahim, Amer Alshengeti, Abdulqader Al-Hebshi, Fayssal Farahat, Ghaya Al Qurainees, Manal Al Saif, Naif Hamdan, Sameera Al Jehani, Walaa Al Mansouri, Mona AlDabbagh

**Affiliations:** 1 Department of Pediatrics, King Abdulaziz Medical City, P.O. Box: 65362, Jeddah, 21556 Saudi Arabia; 2 King Abdullah International Medical Research Centre, Jeddah, Saudi Arabia; 3 King Saud bin Abdulaziz University for Health Sciences, P.O. Box: 65362, Jeddah, 21556 Saudi Arabia; 4 Department of Pediatrics, King Abdulaziz University, Jeddah, Saudi Arabia; 5 Pediatric Department, Saudi German Hospital, Almadinah, Saudi Arabia; 6 Pediatric Department, Cairo University, Cairo, Egypt; 7 Department of Pediatrics, College of Medicine, Taibah University, Almadinah, Saudi Arabia; 8 Department of Pediatrics, Prince Mohammad bin Abdulaziz Hospital, Almadinah, Saudi Arabia; 9 Infection Prevention and Control Program, King Abdulaziz Medical City, Riyadh, Saudi Arabia; 10 Department of Pediatrics, King Abdulaziz Hospital, King Abdullah, International Medical Research Center, P.O. Box: 2477, Al-Ahsa, 31982 Saudi Arabia; 11 Department of Pediatric, King Abdulaziz Hospital, P.O Box: 22421, Jeddah, 21470 Saudi Arabia

**Keywords:** COVID-19, SARS-2, MIS-C coronavirus, Coronaviridae

## Abstract

**Background:**

The Coronavirus Disease 2019 (COVID-19) has affected over 100 million cases worldwide. Children accounted for 1–5% of all cases with less reported symptoms and better prognosis compared to adults. This study aimed to describe the epidemiological characteristics and outcomes of pediatric COVID-19 cases in Saudi Arabia in addition to identifying risk factors associated with disease severity.

**Methods:**

This was a multicenter, cross-sectional retrospective study that included confirmed SARS-CoV-2 infection among pediatric patients (< 14 years) from the time of initial identification in March 2020 to the end of July 2020 in 6 centers across the country. Patients were classified based on clinical severity. Study outcomes included time to recovery, need for invasive ventilation, and mortality. Multivariate logistic regression analysis was conducted to explore factors associated with increased disease severity.

**Results:**

The study enrolled 567 children with (51.5%) were males, and (44.6%) aged from 6 to 14 years old. Asymptomatic patients accounted for 38.98% of the cases: while 319 patients (56%) had mild disease, and 27 patients (4.76%) had moderate-to-severe disease. Only 10 patients (1.76%) required Pediatric Intensive Care Unit admission. The calculated case-fatality was 0.7%. After performing multivariate regression analysis, chronic lung conditions [adjusted OR = 12.73, 95% CI (2.05–79.12)] and decreased red blood cells (RBCs) count [adjusted OR = 2.43, 95% CI (1.09–5.41] were found to be significant predictors for moderate-to-severe disease (*p* = 0.006 and 0.030, respectively).

**Conclusion:**

Most COVID-19 cases in the current study had a benign course of illness and carried an excellent prognosis. Children with chronic lung conditions or low RBCs count are at higher risk to develop moderate-to-severe COVID-19 disease.

**Supplementary Information:**

The online version contains supplementary material available at 10.1186/s12887-021-02959-8.

## Introduction

The Coronavirus Disease 2019 (COVID-19) pandemic was announced on 11 March 2020. Since then, the pandemic has been rapidly changing, affecting communities from all over the world. The disease is caused by the emerging Severe Acute Respiratory Syndrome Coronavirus-2 (SARS-CoV2). It is an enveloped, single-strand, positive-sense RNA virus. Like all the viruses that belong to the family of *Coronaviridae*, it is transmitted by either direct contact or respiratory droplet. Infections caused by viruses from the *Coronaviridae* family mainly affect the respiratory and gastrointestinal systems, causing symptoms that vary from simple common cold or pneumonia to acute respiratory distress syndrome (ARDS), multi-organ failure, or death [1]. Nevertheless, SARS-CoV2 has the highest transmissibility among all CoV viruses with R0 = 2.5 (1.8–3.6) [2].

As reported by the World Health Organization (WHO), and as of March 4, 2021, COVID-19 has affected 219 different countries with more than 114 million confirmed cases; the United States of America being the highest contributing country; of which, 2,543,755 cases died due to severe disease [3]. In Saudi Arabia, the disease has affected many regions of the country with a total of 377,383 confirmed cases and reported overall case-fatality rate of 2.22% [4].

Children account for 1 to 5% of all diagnosed COVID-19 cases and appear to be less likely to report symptoms [5, 6]. Data from Saudi Arabia suggest a pediatric incidence of approximately 8%, most of which are identified as part of contact tracing [7]. Of all pediatric age groups, those under the age of 1 year of age are the most vulnerable for COVID-19 infection [5, 8].

Nowadays, epidemiological data regarding pediatric COVID-19 disease are available, and several studies were done in Saudi Arabia [9, 10]. However, more comprehensive studies are still needed for a better understanding of the disease’s nature, course, risk factors, and outcome. This is a multi-center study aimed to describe the epidemiology, clinical presentation, risk factors, and outcome of COVID-19 disease among children in six secondary-to-tertiary health care centers in Saudi Arabia.

## Materials and methods

### Study design

This was a multicenter, cross-sectional retrospective study. Medical records of pediatric patients (< 14 years) were reviewed to describe epidemiological characteristics and outcomes. The study was conducted in 6 centers: Ministry of National Guard Health Affairs (MNGHA) hospitals in Jeddah, Madinah and Alahsa; King Abdulaziz University hospital- Jeddah; King Abdulaziz hospital- Jeddah, and Saudi German hospital- Madinah.

### Study subjects

Pediatric patients (aged less than 14 years) with confirmed COVID-19 disease based on positive RT-PCR were included. The study duration was between the first of March to the end of July 2020. The exclusion criteria included any patient who had not done SARS CoV-2 PCR or SARS CoV-2 serology test.

Confirmed COVID-19 cases were defined as: patients with suspected COVID-19 that tested positive for SARS CoV-2 as per testing standards, irrespective of clinical signs and symptoms. WHO classification of COVID-19 clinical severity was utilized where COVID-19 cases are placed into one of the following categories: asymptomatic, mild, moderate, severe, or critical disease. Mild disease includes “Symptomatic patients meeting the case definition for COVID-19 without evidence of viral pneumonia or hypoxia. Moderate disease is defined as pneumonia without age-specific signs and symptoms of severe pneumonia. Severe disease refers to pneumonia cases with an age-specific clinical picture of Acute Respiratory Distress Syndrome (ARDS). Critical disease is defined as the presence of acute respiratory distress syndrome, sepsis, or septic shock [11]. Multisystem Inflammatory Syndrome of Children (MIS-C) cases will be defined as per the WHO case definition [12]. WHO case definition of MIS-C was applied in retrospect to the cases presenting with Kawasaki-like Syndrome before its official release.

### Data collection

Data were collected through a web-based generated data collection sheet that was distributed to the contributing centers. Data collection was done through accessing patients’ electronic/paper-based medical records.

The data collection sheet included information related to patients’ demographics, underlying medical condition, clinical manifestations, COVID-19 disease severity classification, laboratory and radiological investigations done, interventions given, and the outcomes (time to recovery, Pediatric or Neonatal Intensive Care Unit (PICU or NICU) admission, need for Positive Pressure Ventilation (PPV), and mortality). Recovery (clinical improvement) was defined as the resolution of symptoms. The need for PPV includes both the use of non-invasive ventilation (C-PAP or Bi-PAP), invasive mechanical ventilation, or Extracorporeal Membrane Oxygenation (ECMO). Mortality related to COVID-19 disease was defined as death within 28 days of the symptoms’ onset.

### Statistical analysis

Statistical Package for Social Sciences (SPSS) version 26.00 was used for data analysis. Categorical data were described in numbers and percentages. Continuous data were described in medians and Interquartile ranges-IQR (Q1-Q3). Descriptive statistics were used to compare between patients with mild disease versus patients with moderate-to-severe disease; continuous variables were compared using Mann–Whitney test, and categorical variables were compared using Chi-square with a statistical significance cutoff set at *p* < 0.05. A primary comparison of outcomes in patients with mild and moderate-to-severe disease was also done.

### Ethical approval

The study proposal was approved by all corresponding institutional review boards (IRBs), the IRB of King Abdullah International Medical Research Center (KAIMRC), the IRB of Ministry of Health, and the IRB of King Abdulaziz University Hospital. All data, both in soft and hard copies, were maintained and saved within MNGHA premises or the specified study centers and accessed by the research team only.

## Results

**Fig. 1:**
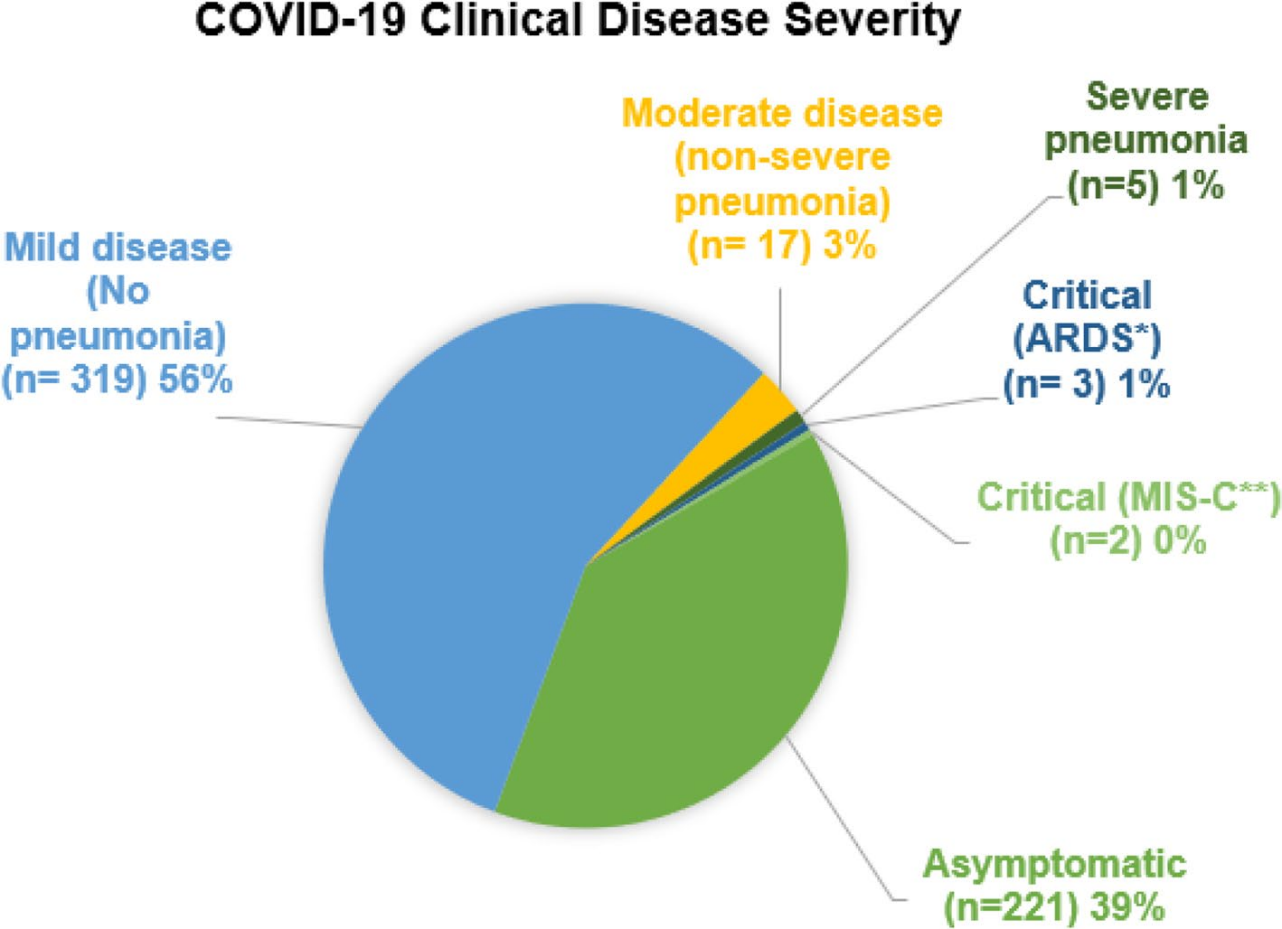
Clinical disease severity of COVID-19 of patients enrolled in the Saudi multicenter study (*n* = 567). * Acute Respiratory Distress Syndrome

From March to July 2020, 567 patients were enrolled in this study from six different centers in Saudi Arabia. More than half of the patients were males (51.5%). Most patients (44.6%, *n* = 253) aged 6–14 years old. Comorbidities upon presentation were identified in 65 patients (11.4%); chronic lung disease was the frequent co-morbidity and was found in 13 patients. Figure 1 shows the clinical severity of COVID-19 disease where most patients (56%, *n* = 319) had mild disease. Some of the cases in the current study were published elsewhere [10].

Table 1 summarizes the general descriptive statistics for the enrolled patients. Patients were categorized into three groups: asymptomatic, mild disease, and moderate to severe disease. No significant difference was found when comparing males to females in terms of the clinical severity of COVID-19 (*p* = 0.91). Of all asymptomatic, the majority were aged 1–6 years old (46.2%). In mild and moderate-to-severe disease categories, most patients aged 6–14 years old (44.2 and 44.4%, respectively). No significant difference was found across all age groups (*p* = 0.051). Less than one-fifth of patients (18.5%) with moderate-to-severe disease had chronic lung disease compared to 1.8% and 2.8% in the asymptomatic and mild disease groups, respectively (*p* < 0.001). Similar findings were found among patients with chronic heart disease where chronic heart conditions were significantly higher in subjects with moderate-to-severe disease compared to the asymptomatic and mild cases (18.5% vs. 2.8% and 1.8% respectively; p < 0.001).

**Table 1:** General descriptive statistics for the enrolled patient

Variables	Total symptomatic patients = 567			*p*-value^¶^
	Asymptomatic	Mild disease	Moderate to severe disease^a^	
	*n* = 221 (%)	*n* = 319 (%)	*n* = 27 (%)	
***Gender***				
*Female*	113 (51.1)	164 (51.4)	15 (55.6)	0.909
*Male*	108 (48.9)	155 (48.6)	12 (44.4)	
***Age groups***				
*Less than 1 month*	0	5 (1.6)	1 (3.7)	0.051
*1–11 months*	19 (8.6)	45 (14.1)	6 (22.2)	
*1–6 years*	102 (46.2)	128 (40.1)	8 (29.6)	
*> 6 years*	100 (45.2)	141 (44.2)	12 (44.4)	
***Co-morbidities***				
*Chronic lung conditions*^b^	4 (1.8)	9 (2.8)	5 (18.5)	0.0001
*Diabetes Mellitus- type 1*	3 (1.4)	2 (0.6)	1 (3.7)	NA
*Hypertension*	0	3 (0.9)	0	NA
*Chronic heart disease*	2 (0.9)	8 (2.5)	5 (18.5)	0.0001
*Human Immunodeficiency Virus (HIV) Infection*	1 (0.5)	2 (0.6)	0	NA
*Epilepsy*	1 (0.5)	3 (0.9)	1 (3.7)	NA
*Hgb and blood-enzymes defects*	1 (0.5)	3 (0.9)	1 (3.7)	NA
*Active malignancy*	4 (1.8)	0	2 (7.4)	NA
*Genitourinary diseases*	2 (0.9)	2 (0.6)	0	NA
*Primary immunodeficiency*	0	0	1 (3.7)	NA
***On immune- modulator/suppressor drugs***	2 (0.9)	2 (0.6)	2 (7.4)	NA
***On chemotherapy***	4 (1.8)	0	1 (3.7)	NA

^¶^ p-value for variables with expected count < 5 in more than 25% of the cells was displayed as NA- (Not Applicable-irrelevant)

^a^ It included severe pneumonia and critical disease (Acute Respiratory Distress Syndrome (ARDS), Sepsis, or Septic Shock)

^b^ It included patients with asthma/hype Active Airway Disease (*n* = 11), ventilator-dependent Cerebral Palsy (*n* = 1), and Bronchiolitis Obliterans (*n* = 1)

Table 2 illustrates the symptomatology upon presentation. Of 345 symptomatic patients, fever, cough, or shortness of breath accounted for 81.79% (*n* = 283). Fever was the most common presenting symptom followed by cough with 59.54% and 48.27%, respectively. Only one patient (0.29%) presented with an altered level of consciousness.

**Table 2:** The reported symptomatology of COVID-19 in the multicenter study^a^

Variables	*n* = 346 (%)
***Fever, cough, or shortness of breath***	283 (81.79)
*Fever*	206 (59.54)
*Cough*	167 (48.27)
*Diarrhea*	71 (20.52)
*Nausea/Vomiting*	64 (18.50)
*Anorexia/Decreased feeding*	55 (15.90)
*Rhinorrhea*	50 (14.45)
*Fatigue/decreased activity*	48 (13.87)
*Shortness of breath*	39 (11.27)
*Headache*	31 (8.96)
*Sore throat*	28 (8.09)
*Myalgia*	20 (5.78)
*Anosmia*	11 (3.18)
*Abdominal pain*	7 (2.02)
*Abnormal movement*	7 (2.02)
*Chest pain*	3 (0.87)
*Ageusia*	3 (0.87)
*Kawasaki-like Syndrome*	2 (0.58)
*Altered level of consciousness*	1 (0.29)

^a^ *221 patients (39%) were asymptomatic at presentation*

Table 3 shows the laboratory workup done for symptomatic patients (*n* = 345). Hemoglobin level was significantly lower in the moderate-to-severe disease group compared to mild disease group (median 10.8 vs. 12.60 respectively; *p* = 0.001). Similar findings were found regarding Red-Blood-Cells (RBCs) count (4.21 vs. 4.69 respectively; *p* = 0.001). On the other hand, International Normalized Ratio (INR) (media*n* = 1.2 vs. 1.1; *p* = 0.004), D-dimer (median = 1.08 vs. 0.60; *p* = 0.018), C-Reactive Protein (CRP) (median = 5.50 vs. 3.0; *p* = 0.001), and Erythrocyte Sedimentation Rate (ESR) (median = 16.50 vs. 5.0; *p* = 0.026) levels were found significantly higher in the moderate-to-severe disease group compared to the mild disease group. Additional file 1 shows case counts per variables that were analyzed in Table 3.

**Table 3:** Investigations done to the symptomatic patients in the multicenter study

Variables	Total symptomatic patients = 346		*p-*value^¶^
	Mild disease	Moderate to severe disease^a^	
	*n* = 319	*n* = 27	
*Median* ***Hgb in gm/dL*** *(Q1-Q3)*	12.60 (11.60–13.5)	10.80 (8.90–12.7)	0.001
*Median* ***RBCs × 10***^***12***^***/L*** *(Q1-Q3)*	4.69 (4.31–5.07)	4.21 (3.69–4.65)	0.001
*Median* ***Neutrophils ×10***^***9***^***/L*** *(Q1-Q3)*	2.68 (1.35–5.27)	2.99 (1.31–6.2)	0.754
*Median* ***Lymphocytes × 10***^***9***^***/L*** *(Q1-Q3)*	3.03 (1.79–5.16)	3.00 (3.01–4.26)	0.838
*Median* ***Basophils ×10***^***9***^***/L*** *(Q1-Q3)*	0.04 (0.02–0.09)	0.05 (0.02–0.08)	0.847
*Median* ***Eosinophils ×10***^***9***^***/L*** *(Q1-Q3)*	0.15 (0.08–0.35)	0.1 (0.03–0.208)	0.119
*Median* ***Monocytes ×10***^***9***^***/L*** *(Q1-Q3)*	0.71 (0.48–1.18)	0.76 (0.49–1.87)	0.569
*Median* ***Platelets ×10***^***9***^***/L*** *(Q1-Q3)*	305 (298–386)	315 (146.50–399.5)	0.510
*Median* ***Platelets/lymphocyte ratio*** *(Q1-Q3)*	93.27 (61.17–157.61)	87.55 (60.29–147.42)	0.656
*Median* ***Neutrophil/lymphocyte ratio*** *(Q1-Q3)*	0.86 (0.41–1.81)	1.12 (0.46–2.43)	0.588
*Median* ***PTT/APTT in seconds*** *(Q1-Q3)*	32.80 (31–34)	33.2 (31.8–41.56)	0.189
*Median* ***INR in seconds*** *(Q1-Q3)*	1.1 (1–1.2)	1.2 (1.1–1.4)	0.004
*Median* ***Fibrinogen in g/L*** *(Q1-Q3)*	3.07 (1.3–3.59)	3.62 (2.55–8.63)	0.808
*Median* ***D-dimer in mg/L*** *(Q1-Q3)*	0.6 (0.28–1.05)	1.08 (0.61–5.89)	0.018
*Median* ***CK in IU/L*** *(Q1-Q3)*	86 (44.25)	46 (115)	0.452
*Median* ***SrCr in umol/L*** *(Q1-Q3)*	37.5 (30.13–46)	35 (16–43)	.0133
*Median* ***BUN in mmol/L*** *(Q1-Q3)*	3.80 (2.90–6.1)	4.5 (3.35–5.23)	0.445
*Median* ***ALT in U/L (****(Q1-Q3)*	20 (14–31.5)	21 (15–37)	0.471
*Median* ***AST in U/L*** *(Q1-Q3)*	32.50 (25–43.75)	32 (21.5–37)	0.767
*Median* ***CRP in mg/L*** *(Q1-Q3)*	3.0 (1–5.2)	5.5 (3.14–66.15)	0.001
*Median* ***ESR in mm/hr*** *(Q1-Q3)*	5 (2–10)	16.5 (11.75–55.75)	0.026
***Chest x-ray (CXR) findings***			
*Not done*	226 (70.8%)	0	NA
*Normal*	87 (27.3%)	5 (18.5%)	
*Lobar infiltration*	4 (1.3%)	8 (29.6%)	
*Broncho-pneumonic infiltrations*	1 (0.3%)	8 (29.6)	
*Bilateral consolidation*	1 (0.3%)	6 (2.2)	
***Chest CT scan***			NA
*Not Done*	317 (99.4%)	25 (92.6%)	
*Done (Normal)*	2 (0.6%)	0	
*Done (abnormal)*	0	2 (7.4%)	

^¶^ p-value for categorical variables with expected count < 5 in more than 25% of the cells was displayed as NA- (Not Applicable-irrelevant). P-value for scale variables was calculated using Mann-Whitney U test

^a^ It included severe pneumonia and critical disease (Acute Respiratory Distress Syndrome (ARDS), Sepsis, or Septic Shock

Only ten patients required Intensive Care Unit (ICU) admission; Nine to the PICU and one to the NICU. The profile of ICU patients and the need for ventilatory support is summarized in Table 4; six patients required invasive mechanical ventilation, and 4 patients required noninvasive CPAP. The median and the (IQR) of duration on invasive ventilation were 5 (2–22) days.

**Table 4:** Clinical profile of patients who required Intensive Care Unit (ICU) admission

Variables	*n* = 10
*Types of ventilatory support*	
*Invasive pressure control*	2 (20%)
*Invasive pressure support*	1 (10%)
*Invasive SIMV*	1 (10%)
*Invasive volume control*	2 (20%)
*Non-invasive C-PAP*	4 (40%)
*Median Duration on Invasive Ventilation*^a^ *(Q1-Q3)*	5 (2–22)

^a^One patient was still ventilated beyond the study period

Table 5 demonstrates treatment modalities, complications, and outcomes of the included COVID-19 patients; no side effects from the treatment modalities were noticed. From the mild disease group, 94% of the patients received supportive care only in comparison to 40% in moderate to severe disease groups (*p* = 0.001). All patients with the mild disease successfully recovered compared to 85.2% in the moderate-to-severe group. No difference was found regarding time to symptoms resolution in days between mild [median = 6, IQR = (2–9)] and moderate-to severe [median = 8, IQR = (4–9)] disease groups (*p* = 0.345).

**Table 5:** Treatment, complication, and Outcomes of COVID-19 in terms of disease severity

Variables	Total symptomatic patients = 346		*p*-value^¶^
	Mild disease	moderate to severe disease^a^	
	*n* = 319	*n* = 27	
***Supportive Care Only*** *(*e.g.*, hydration, temperature control,* etc.*)*	301 (94.4)	11 (40.7)	0.0001
Antimicrobials*:			
*No antimicrobial used*	302 (94.7)	17 (63)	0.0001
*Hydroxychloroquine/Chloroquine*	1 (0.3)	3 (11.1)	NA
*Hydroxychloroquine/Chloroquine + any macrolide*	7 (2.2)	5 (18.5)	0.0001
*Azithromycin alone*	9 (2.8)	2 (7.4)	0.192
***Anticoagulant therapy***			
*Unfractionated Heparin*	1 (0.3)	0	NA
***Antivirals used:***			
*No antiviral used*	319 (100)	23 (85.2)	NA
*Umifenovir*	0	0	NA
*Favipiravir*	0	3 (11.1)	NA
*Interferon β-1a*	0	1 (3.7)	NA
***Biologicals used:***			
*Tocilizumab*	0	0	NA
***Convalescent Plasma Therapy***	0	1 (3.7)	NA
***Complications***			
*Myocarditis*	0	1 (3.7)	NA
*Massive pericardial effusion*	0	1 (3.7)	
***Outcome***			
Recovery	319 (100)	23 (85.2)	NA
Death	0	4 (14.8)	

^¶^ p-value for variables with expected count < 5 in more than 25% of the cells was displayed as NA- (Not Applicable-irrelevant)

*overall p-value for all variables is NA

^a^ It included severe pneumonia and critical disease (Acute Respiratory Distress Syndrome (ARDS), Sepsis, or Septic Shock)

Only two complications due to COVID-19 with recovery were observed in the study. One patient aged 12 years and was a known case of epidermolysis bullosa and cardiomyopathy. He presented with fever, cough, and mild shortness of breath with evidence of myocarditis on echocardiography. He was treated in the general ward and recovered after receiving Intravenous Immunoglobulin (IVIG) and Dexamethasone. The other patient aged 6 years and was medically and surgically free. He presented with MIS-C and evidence of bilateral consolidation in chest x-ray and cardiac tamponade on echocardiography. The patient required PICU admission and received IVIG, Dexamethasone, Favipiravir, Hydroxychloroquine, and Azithromycin. He was later discharged in good condition after being hospitalized for 10 days.

A total of four patients (14.8%) in the moderate to severe group died with an overall case-fatality rate of 0.7%. One patient aged 17 months and was having severe aplastic anemia and pulmonary tuberculosis on treatment. He presented with severe pneumonia that was complicated by ARDS; he subsequently died from septic shock. The second patient aged 4 months and was under investigation for inborn error of metabolism. He was tested positive for COVID-19, but his death was most likely attributed to his metabolic disease. The third patient aged 6 months and was diagnosed with spinal muscular atrophy (SMN1-EXON7 deletion); he was severely hypotonic and was on limited code status before developing COVID-19. The fourth patient aged 11 years and was medically and surgically free. He presented with a picture of MIS-C. The patient later developed ARDS and subsequently died.

Chronic lung conditions, chronic heart conditions, hemoglobin level, RBCs count, and CRP level were entered into a multivariate regression analysis to identify risk factors/predictors for moderate-to-severe COVID-19 (Table 6). After controlling for potential confounders, only chronic lung conditions [adjusted OR = 12.72, 95% CI (2.05–79.12), *p* = 0.006] and low RBCs count [adjusted OR = 0.41, 95% CI (0.19–0.92), *p* = 0.30] were significant predictors for moderate-to-severe COVID-19 in the model.

**Table 6:** Multivariate analyses for risk factors/predictors associated with moderate-to-severe COVID-19 in children^a^

Variables	Symptomatic patients		Crude Odds Ratio^b^ (CI)	Adjusted Odds Ratio^b^ (CI)	*p*-value
	Mild disease *n* = 91	Moderate-to-severe disease *n* = 25			
***Chronic lung conditions***^**c**^	3 (3.30%)	4 (16%)	5.59 (1.16–26.88)	12.72 (2.05–79.12)	0.006
***Chronic heart disease***	5 (5.49%)	5 (20%)	4.30 (1.14–16.29)	2.86 (0.65–12.57)	0.165
*Median* ***Hgb in gm/dL*** *(Q1-Q3)*	12.7 (11.7–13.4)	10.4 (8.75–12.9)	1.33 (1.1–1.61)	1.19 (0.92–1.56)	0.193
*Median* ***Red-Blood-Cells count ×10***^***12***^***/L*** *(Q1-Q3)*	4.7 (4.4–5.1)	4.21 (3.48–4.72)	2.7 (1.49–4.76)	2.44 (1.09–5.26)	0.030
*Median* ***CRP in mg/L*** *(Q1-Q3)*	3 (0.9–5.8)	5.5 (3.14–66.15)	1.009 (1.002–1.02)	1 (0.998–1.015)	0.129

^a^Multiple cases had missing values given the retrospective nature of the study. The analysis was limited to the cases where all of the above-mentioned variables were available.

^b^Odd ratios for Hgb and RBCs were inverted for clarity.

^c^It included patients with asthma/hyper-active airway disease (*n* = 11), ventilator-dependent Cerebral Palsy (*n* = 1), and Bronchiolitis Obliterans (*n* = 1)

## Discussion

This multi-center study described the epidemiology, clinical presentation, and outcomes of pediatric COVID-19 in Saudi Arabia. The majority (95%) of the patients were asymptomatic or had mild disease. Almost half of the patients were >  6 years of age, including those with moderate-to-severe disease. Most patients received supportive care only; few patients required admission to the ICU, and the fatality rate was very low. The study also found that having an underlying chronic lung condition and reduced RBCs are risk factors for developing moderate-to-severe disease.

The epidemiology of COVID-19 in Saudi Arabia and the Gulf region was described in multiple studies. For example, Al Yazidi et al. found in a multicenter cohort from Oman that 68% of the documented cases (*n* = 56) had uncomplicated COVID-19; of which 13% were admitted to the PICU. Only one patient required invasive mechanical ventilation, and no mortality was reported [13]. Alharbi et al. conducted a comprehensive, single-center study on COVID-19 in children from Riyadh; 742 patients with positive SARS-2 PCR were included, of which 60% were > 5 years of age. 71 patients (9.6%) required hospitalization while 12 patients required PICU transfer with only three reported deaths [9].

Data from worldwide reports were included in a systematic review on childhood COVID-19 with a sample size of 1124 cases; severe/critical disease occurred in < 3.5% of the cases, with a single reported death [14]. Similarly, a recent multicenter report from Europe suggested that asymptomatic cases account for 16% of the identified cases with 8% severe diseases and a fatality rate of 0.69% [15]. Another study published from China suggested that 86% of all early COVID-19 infections remained undiagnosed and these cases may have had a major role in infection transmission [16].

Although it did not reach statistical significance, this study suggests that almost half of the confirmed COVID-19 cases, including those with more severe disease, were more than 6 years of age. This finding was in agreement with other Saudi data [9]. Nonetheless, most international data suggested that those less than 1 year of age were the most vulnerable group [17, 18].

The current study found a similarly low rate of severe disease and case fatality rate. Preliminary evidence suggests that children become infected with SARS-CoV-2 just like adults; however, they are less likely to express symptoms or develop severe disease and accordingly are less likely to die. This could be attributed to the differences in the function of the adult immune system and/or differences in the cellular receptor expression among patients with SARS-CoV2- such as Angiotensin Converting Enzyme-2 (ACE-2) receptor [8, 19]. Another proposed explanation would be related to repeated viral exposures in children which strengthens the immune system to fight SARS CoV-2 [20]. On the other hand, the high rate of asymptomatic cases in the current study could be attributed to the meticulous contact tracing that was undertaken in the country. Nonetheless, the role of children in infection transmission escalating remains unclear.

The role of underlying medical conditions as risk factors for severe COVID-19 in children was investigated in few studies. Graff et al. reported that immunocompromising conditions, gastrointestinal conditions, diabetes, and asthma are predictors for hospital admission. In addition, asthma and gastrointestinal conditions were associated with increased odds of requiring respiratory support. The current study found that those with underlying lung conditions, for which the majority had asthma/hyperactive airway disease, were more likely to develop moderate-to-severe disease. However, we could not demonstrate any further association with any other comorbidities. This could be explained by the very small number of cases with such comorbidities [17].

A meta-analysis also looked at the effect of anemia and iron metabolism in COVID-19 and found that severe cases, compared to moderate COVID-19 cases, had significantly lower hemoglobin and RBCs count, and higher ferritin level. It is hypothesized that the innate immune system in the acute phase of infection may aim to decrease iron bioavailability in order to prevent an expanding viral load. This results in the activation of hepcidin, sequestration of intracellular iron, increased ferritin levels, drop-in hemoglobin level, and resultant hypoxia [21]. These findings are similar to ours where moderate-to-severe disease group had significantly lower hemoglobin and RBCs in comparison to the mild group. Nevertheless, after performing multivariate analysis, only RBCs count was found to be a significant predictor for moderate-to-severe COVID-19.

In addition, the prognostic role of elevated inflammatory markers and Prothrombin time was reported in many studies. A meta-analysis conducted by Elshazli et al. reported that elevated CRP, procalcitonin, ESR, and prothrombin time were significantly associated with increased odds of severe disease progression [22]. These findings were compatible with ours in the univariate analysis. Yet, after performing multivariate regression analysis, none of the inflammatory and coagulation markers showed a significant increase in the odds for moderate-to-severe disease.

Gracia Salido et al. reported 74 patients with PICU admissions due to severe COVID-19; 60% fulfilled the criteria of MIS-C diagnosis. Lopinavir-ritonavir was used in 40% of patients. No difference was noticed in the outcome if antiviral treatment was given [23]. In the current study, two patients were diagnosed with MIS-C. The first patient was admitted to the PICU and received extensive management and recovered after 10 days. The second patient developed severe ARDS and died. In addition, the use of antivirals was very limited to certain cases and no effect on the outcome was observed; all the patients who received antiviral treatment died.

## Conclusion

Most COVID-19 cases in the current study had a benign course of illness and carried an excellent prognosis, which is comparable to most studies worldwide. Chronic lung disease and decreased RBCs were found to be significant predictors for moderate-to-severe COVID-19 presentation. No recommendation could be made regarding the use of antivirals and immunomodulatory therapy due to the low number of severe cases and mortality. There is still a need for further studies identifying the contributing risk factors and best therapeutic modalities in pediatric COVID-19.

### Limitations

Although the current study provided a sufficient sample size, it was limited by the retrospective design, the limited number of patients with moderate-to-severe disease, and the low mortality rate. This led to major constraints in the analysis due to missing laboratory investigations and diagnostic workup. In addition, the study could not assess the effect of using antivirals or immunomodulatory agents on the outcome. Future large prospective studies examining risk factors for severe COVID-19 in children are warranted to understand the full picture of pediatric COVID-19. In addition, more robust data on the best treatment modalities are strongly indicated.

## Supplementary Information

**Additional file 1.**

## Data Availability

The datasets used and/or analyzed during the current study are available from the corresponding author on reasonable request.

## Abbreviations

COVID-19: The Coronavirus Disease 2019
SARS-CoV2: Severe Acute Respiratory Syndrome Coronavirus-2
WHO: World Health Organization
MNGHA: Ministry of National Guard Health Affairs
ARDS: Acute Respiratory Distress Syndrome
MIS-C: Multisystem inflammatory syndrome of Children
PICU: Pediatric Intensive Care Unit
NICU: Neonatal Intensive Care Unit
PPV: Positive Pressure Ventilation
ECMO: Extracorporeal Membrane Oxygenation
SPSS: Statistical Package for Social Sciences
IQR: interquartile ranges (Q1-Q3)
IRB: Institutional Review Board
KAIMRC: King Abdullah International Medical Research Center
RBCs: Red Blood Cells
INR: International Normalized Ratio
CRP: C-Reactive Protein
ESR: Erythrocyte Sedimentation Rate
IVIG: Intravenous Immunoglobulin
OR: Odds ratio
CI: Confidence interval
ACE-2: Angiotensin-Converting Enzyme-2.
