# Supplementary material for: Epidemiology, clinical characteristics and risk factors of COVID-19 among children in Saudi Arabia: a multicenter chart review study
Source: BMC Pediatr. 2022 Feb 12;22:86. doi: 10.1186/s12887-021-02959-8 (PMC8840071; doi:10.1186/s12887-021-02959-8)
Supplement: Supplementary file 1 — Additional file 1. [file 12887_2021_2959_MOESM1_ESM.docx]

| **Additional file 1:** Case counts for investigations done to the symptomatic patients | | |
| --- | --- | --- |
|  | | |
| **Variables** | Total symptomatic patients= 356 | |
|  | Valid values in mild disease group | Valid values moderate  to a severe disease group^!^ |
|  | n=319 | n=27 |
| ***Hgb*** *(gm/dL)* | 135 (42.3%) | 27 (100%) |
| ***RBCs*** *(x10^12^/L)* | 134 (42%) | 27 (100%) |
| ***Neutrophils*** *(x10^9^/L)* | 127 (39.8 %) | 26 (96.2%) |
| ***Lymphocytes*** *(x10^9^/L)* | 133 (41.6%) | 26 (96.2%) |
| ***Basophils*** *(x10^9^/L)* | 99 (31%) | 20 (74%) |
| ***Eosinophils*** *(x10^9^/L)* | 122 (38.2%) | 24 (88.9%) |
| ***Monocytes*** *(x10^9^/L)* | 132 (41.3%) | 24 (88.9%) |
| ***Platelets*** *(x10^9^/L)* | 131 (41%) | 25 (92.59%) |
| ***Platelets/lymphocyte ratio*** | 127 (39.8%) | 25 (92.59%) |
| ***Neutrophil/lymphocyte ratio*** | 127 (39.8%) | 26 (96.2 %) |
| ***PTT/APTT (****in seconds)* | 51 (15.9%) | 17 (62.9%) |
| ***INR*** *(in seconds)* | 54 (16.9%) | 20 (74%) |
| ***Fibrinogen*** *(g/L)* | 4 (1.2%) | 8 (29.65 %) |
| ***D-dimer*** *(mg/L)* | 37 (11.5%) | 16 (59.25%) |
| ***CK*** *(IU/L)* | 17 (5.3 %) | 6 (22.2%) |
| ***SrCr*** *(umol/L)* | 100 (31.3%) | 19 (70%) |
| ***BUN*** *(mmol/L)* | 119 (37.3%) | 22 (81.4 %) |
| ***ALT*** *(U/L)* | 84 (26.3%) | 21 (77.7%) |
| ***AST*** *(U/L)* | 84 (26.3%) | 21 (77.7%) |
| ***CRP*** *(mg/L)* | 99 (31%) | 25 (92.5 %) |
| ***ESR*** *(mm/hr)* | 38 (11.9%) | 6 (22.2 %) |
|  | | |
